# Use of *Haemophilus influenzae* Type b–Containing Vaccines Among American Indian and Alaska Native Infants: Updated Recommendations of the Advisory Committee on Immunization Practices ― United States, 2024

**DOI:** 10.15585/mmwr.mm7336a4

**Published:** 2024-09-12

**Authors:** Jennifer P. Collins, Jamie Loehr, Wilbur H. Chen, Matthew Clark, Veronica Pinell-McNamara, Lucy A. McNamara

**Affiliations:** ^1^Division of Bacterial Diseases, National Center for Immunization and Respiratory Diseases, CDC; ^2^Cayuga Family Medicine, Ithaca, New York; ^3^University of Maryland School of Medicine, Baltimore, Maryland; ^4^Alaska Area Native Health Service, Indian Health Service, Anchorage, Alaska.

**Summary**

**What is already known about this topic?**

*Haemophilus influenzae* type b (Hib) vaccination with a monovalent Hib conjugate vaccine consisting of Hib capsular polysaccharide (polyribosylribitol phosphate [PRP]) conjugated to the outer membrane protein complex of *Neisseria meningitidis* serogroup B (PRP-OMP [PedvaxHIB]) has historically been preferred for American Indian and Alaska Native (AI/AN) infants to provide earlier protection in these populations at increased risk for invasive Hib disease.

**What is added by this report?**

On June 26, 2024, the Advisory Committee on Immunization Practices recommended that hexavalent Vaxelis (diphtheria and tetanus toxoids and acellular pertussis, inactivated poliovirus, Hib conjugate, and hepatitis B vaccine [DTaP-IPV-Hib-HepB]) should be included with monovalent PRP-OMP in the preferential recommendation for AI/AN infants based on the Hib component.

**What are the implications for public health practice?**

A primary Hib vaccination series consisting of monovalent PRP-OMP or DTaP-IPV-Hib-HepB is preferred for AI/AN infants.

## Abstract

Invasive *Haemophilus influenzae* type b (Hib) disease is a serious bacterial infection that disproportionally affects American Indian and Alaska Native (AI/AN) populations. Hib vaccination with a monovalent Hib conjugate vaccine consisting of Hib capsular polysaccharide (polyribosylribitol phosphate [PRP]) conjugated to outer membrane protein complex of *Neisseria meningitidis* serogroup B, PRP-OMP (PedvaxHIB, Merck and Co., Inc.) has historically been preferred for AI/AN infants, who are at increased risk for invasive Hib disease, because it provides substantial protection after the first dose. On June 26, 2024, CDC’s Advisory Committee on Immunization Practices (ACIP) recommended that a hexavalent, combined diphtheria and tetanus toxoids and acellular pertussis (DTaP), inactivated poliovirus (IPV), Hib conjugate, and hepatitis B (HepB) vaccine, DTaP-IPV-Hib-HepB (Vaxelis, MSP Vaccine Company) should be included with monovalent PRP-OMP in the preferential recommendation for AI/AN infants because of the PRP-OMP Hib component. A primary Hib vaccination series consisting of either 1) monovalent PRP-OMP (2-dose series at ages 2 and 4 months) or 2) DTaP-IPV-Hib-HepB (3-dose series at ages 2, 4, and 6 months) is preferred for AI/AN infants. DTaP-IPV-Hib-HepB is only indicated for use in infants at ages 2, 4, and 6 months and should not be used for the booster doses of Hib, DTaP, or IPV vaccines. For the booster dose of Hib vaccine, no vaccine formulation is preferred for AI/AN children; any Hib vaccine (except DTaP-IPV-Hib-HepB) should be used. This report summarizes evidence considered for these recommendations and provides clinical guidance for the use of Hib-containing vaccines among AI/AN infants and children.

## Introduction

Before 1985, *Haemophilus influenzae* type b (Hib) was the leading cause of bacterial meningitis and a common cause of other invasive infections among U.S. children aged <5 years (*1*). After the introduction of effective Hib vaccines,* the incidence of invasive Hib disease among U.S. children aged <5 years declined >99% (*1*). Before and since the introduction of Hib vaccines, American Indian and Alaska Native (AI/AN) populations have been disproportionately affected by invasive Hib disease. The five Hib conjugate vaccines currently licensed and available in the United States consist of Hib capsular polysaccharide (polyribosylribitol phosphate [PRP]) conjugated to either outer membrane protein complex of *Neisseria meningitidis* serogroup B (PRP-OMP) or tetanus toxoid (PRP-T).

Hib vaccination with monovalent PRP-OMP (PedvaxHIB, Merck and Co., Inc.) has historically been preferred for AI/AN infants based on the epidemiology of invasive Hib disease among AI/AN populations and the differing immunogenicity of Hib vaccines. Before routine Hib vaccination began, the incidence of Hib meningitis peaked at a younger age (4–6 months) among AI/AN infants than among other U.S. infant populations (6–7 months) (*2*). Monovalent PRP-OMP provides a protective antibody response after the first dose, recommended at age 2 months, whereas PRP-T vaccines do not (*1*). For this reason, Hib vaccination with monovalent PRP-OMP has been preferred for AI/AN infants, to provide earlier protection against invasive Hib disease.

DTaP-IPV-Hib-HepB (Vaxelis, MSP Vaccine Company^†^) is a hexavalent, combined diphtheria and tetanus toxoids and acellular pertussis (DTaP), inactivated poliovirus (IPV), Hib conjugate, and hepatitis B (HepB) vaccine that contains PRP-OMP as the Hib component. However, DTaP-IPV-Hib-HepB has not previously been included in the preferential recommendation for AI/AN infants, because the dose of PRP-OMP is lower than that in PedvaxHIB, and post–first dose immunogenicity data for DTaP-IPV-Hib-HepB were not previously available (*3*).

## Methods

Five Hib conjugate vaccines are currently licensed and available in the United States.^§^ During December 2023–June 2024, CDC’s Advisory Committee on Immunization Practices (ACIP) Meningococcal/Hib Vaccines Work Group (Work Group) held conference calls to review Hib disease epidemiology among AI/AN populations and new data comparing immunogenicity of DTaP-IPV-Hib-HepB and monovalent PRP-OMP. The policy question under consideration was “Should DTaP-IPV-Hib-HepB (Vaxelis) be included with monovalent PRP-OMP (PedvaxHIB) in the preferential recommendation for American Indian and Alaska Native infants based on the Hib component?” In January 2024, CDC’s National Center for Immunization and Respiratory Diseases and Office of Tribal Affairs and Strategic Alliances held a listening session to obtain input from tribal communities and Indian Health Service staff members regarding the policy question. To guide deliberations, ACIP used the Evidence to Recommendations (EtR) Framework and considered the importance of Hib disease among AI/AN populations within the following domains: as a public health problem; benefits and harms of DTaP-IPV-Hib-HepB among AI/AN infants; the values of the target population; acceptability; resource use; equity; and feasibility.^¶^ ACIP evaluated the available evidence on prespecified outcomes, each with ranked importance, using the Grading of Recommendations, Assessment, Development and Evaluation (GRADE) approach (*4*): invasive Hib disease (important), post–dose 1 immunity (critical), post–primary series immunity (important), and serious adverse events (critical).**

## Summary of Evidence for Use of DTaP-IPV-Hib-HepB Among American Indian and Alaska Native Infants

The Work Group determined that considerations within each domain of the EtR Framework supported inclusion of DTaP-IPV-Hib-HepB in the preferential recommendation for AI/AN infants. The GRADE assessment of benefits and harms is summarized here.

No data were available to assess the outcome of invasive Hib disease. For the remaining outcomes, the body of evidence comprised data from one phase IV, prospective, open-label randomized controlled clinical trial comparing immunogenicity and safety of DTaP-IPV-Hib-HepB vaccine versus monovalent PRP-OMP vaccine among 333 Navajo Nation and Alaska Native infants (*5*). The clinical trial enrolled healthy infants born at ≥35 weeks’ gestational age, aged 42–90 days at the time of first vaccination, and identified as AI/AN by the parent or legally authorized representative. Infants were randomized to a primary series of DTaP-IPV-Hib-HepB (administered at ages 2, 4, and 6 months) or monovalent PRP-OMP (administered at ages 2 and 4 months). Anti-Hib immunoglobulin G (IgG) antibody levels were measured before vaccination and on days 30, 120, and 150 after the first vaccine dose. Constrained longitudinal data analysis was used to evaluate the anti-Hib IgG geometric mean concentration (GMC) ratio (DTaP-IPV-Hib-HepB versus monovalent PRP-OMP) 30 days after dose 1; the investigators’ prespecified noninferiority criterion for the lower bound of the 95% CI was >0.67. Safety monitoring for serious adverse events^††^ was conducted on days 0, 30, 60, 120, and 150 after the first vaccine dose.

### Post–Dose 1 Immunity

The anti-Hib IgG GMC ratio (DTaP-IPV-Hib-HepB versus monovalent PRP-OMP) 30 days after receipt of dose 1 modeled by constrained longitudinal data analysis was 1.03 (95% CI = 0.75–1.41) and met the noninferiority criterion. The proportion of infants with anti-Hib concentration above the putative correlate of short-term protection (≥0.15 *μ*g/mL) 30 days after receipt of dose 1 was similar in the DTaP-IPV-Hib-HepB group (75.7%) and the monovalent PRP-OMP group (71.2%; p = 0.39). The overall level of certainty for this outcome was moderate.

### Post–Primary Series Immunity

The proportion of infants with anti-Hib concentration above the putative correlate of long-term protection (≥1.0 *μ*g/mL) 150 days after receipt of dose 1 was higher in the DTaP-IPV-Hib-HepB group (3-dose primary series) (83.6%) than in the monovalent PRP-OMP group (2-dose primary series) (71.8%; p = 0.03). The overall level of certainty for this outcome was moderate.

### Serious Adverse Events

The frequency of serious adverse events was similar in the DTaP-IPV-Hib-HepB group (5.4%) and the monovalent PRP-OMP group (7.2%; p = 0.49). The most common serious adverse event was acute respiratory infection (21 of 25; 84%). No serious adverse events were deemed related to study participation. The overall level of certainty for this outcome was moderate.

## Recommendations for Use of Hib-Containing Vaccines Among American Indian and Alaska Native Infants and Children

DTaP-IPV-Hib-HepB is included with monovalent PRP-OMP in the preferential recommendation for American Indian and Alaska Native infants. The basis for this recommendation is the Hib component of PRP-OMP.

### Hib Vaccine Primary Series

A primary Hib vaccination series consisting of either 1) monovalent PRP-OMP (2-dose series at ages 2 and 4 months) or 2) DTaP-IPV-Hib-HepB (3-dose series at ages 2, 4, and 6 months) is preferred over other Hib vaccine formulations for AI/AN infants. If the first Hib vaccine dose is delayed by >1 month, the recommended catch-up schedule should be followed.^§§^

### Booster Dose

DTtaP-IPV-Hib-HepB is only indicated for use in infants aged 2, 4, and 6 months and should not be used for the booster doses of Hib, DTaP, or IPV vaccines (Table 1) (*3*). For the booster dose of Hib vaccine, no vaccine formulation is preferred for AI/AN children (*1*); any Hib vaccine except DTaP-IPV-Hib-HepB should be used. In clinics caring for AI/AN children, stocking monovalent PRP-OMP for the Hib booster dose would maintain parent, guardian, and provider flexibility to choose this vaccine for the primary Hib series. Stocking a PRP-T vaccine for the Hib booster dose also is an option (Table 2). A group of 124 AI/AN infants who took part in a phase 3 study of a DTaP-IPV-Hib-HepB primary series with a heterologous Hib booster (PRP-T) demonstrated a robust immune response (*6*).

**TABLE 1:** Child and adolescent immunization schedule for components of Vaxelis vaccine, by age — United States, 2024

Vaccine	Recommended age for receipt of vaccine dose				
	Primary series			Booster doses	
	1st dose	2nd dose	3rd dose	1st booster dose*	2nd booster dose*
HepB^ⴕ^	Birth^§^	1–2 mos	6–18 mos^¶^	NA	NA
DTaP	2 mos	4 mos	6 mos	15–18 mos	4–6 yrs
Hib	2 mos	4 mos	6 mos**	12–15 mos	NA
IPV	2 mos	4 mos	6–18 mos	4–6 yrs	NA

**Abbreviations**: DTaP = diphtheria and tetanus toxoids and acellular pertussis; HepB = hepatitis B; Hib = *Haemophilus influenzae* type b; IPV = inactivated poliovirus; NA = not applicable; Vaxelis = hexavalent DTaP-IPV-Hib-Hep B vaccine.

* Vaxelis is not indicated for the booster doses of DTaP, Hib, or IPV.

^†^ Administration of 4 doses is permitted when a combination vaccine containing HepB is used after the birth dose. Clinicians should refer to previously published recommendations for additional guidance based on maternal hepatitis B infection status, infant birthweight, and vaccine manufacturer. https://pubmed.ncbi.nlm.nih.gov/29939980/

^§^ Vaxelis is not indicated.

^¶^ For an adequate immune response, the last dose of HepB should be given at age ≥24 weeks.

** Not applicable for monovalent PRP-OMP (PedvaxHIB).

**TABLE 2:** Characteristics of licensed and available *Haemophilus influenzae* type b–containing vaccines — United States, 2024

Vaccine product	Trade name	Recommended ages for administration	
		Primary series	Booster dose
**Monovalent vaccines**			
PRP-OMP*	PedvaxHIB	2 and 4 mos	12–15 mos
PRP-T	ActHIB	2, 4, and 6 mos	12–15 mos
PRP-T	Hiberix	2, 4, and 6 mos	12–15 mos
**Combination vaccines**			
DTaP-IPV/Hib^ⴕ^	Pentacel	2, 4, and 6 mos	12–15 mos
DTaP-IPV-Hib-HepB^§^	Vaxelis	2, 4, and 6 mos	Not indicated^¶^

**Abbreviations:** DTaP = diphtheria and tetanus toxoids and acellular pertussis; HepB = hepatitis B; Hib = *Haemophilus influenzae* type b; IPV = inactivated poliovirus; OMP = outer membrane protein complex of *Neisseria meningitidis* serogroup B; PRP = polyribosylribitol phosphate; T = tetanus toxoid.

* Contains 7.5 *μ*g PRP and 125 *μ*g OMP.

^†^ Hib component is PRP-T.

^§^ Hib component is PRP-OMP. Contains 3 *μ*g PRP and 50 *μ*g OMP.

^¶^ Not indicated for use as a booster dose. A different Hib-containing vaccine should be used.

If DTaP-IPV-Hib-HepB is inadvertently administered for the booster dose or doses of Hib, DTaP, or IPV vaccines, the dose of the corresponding component does not need to be repeated when the proper spacing of doses is maintained. For additional guidance for use of DTaP-IPV-Hib-HepB in infants, clinicians should refer to previously published recommendations (*3*).

## Reporting of Vaccine Adverse Events

Adverse events that occur in a patient after vaccination should be reported to the Vaccine Adverse Event Reporting System (VAERS), even if it is uncertain whether the vaccine caused the event. Instructions for reporting to VAERS are available at https://vaers.hhs.gov/reportevent.html or by calling 800–822–7967. Vaccine adverse event reports originating from the Indian Health Service (IHS) I/T/U (IHS Federal/Tribal/Urban) system of care should include the abbreviation “IHS” in item #26 of the VAERS report form to facilitate tracking such events among the IHS service population.

## Footnotes

* Hib polysaccharide vaccines were first introduced in 1985, and conjugate vaccines in 1987.

† MSP Vaccine Company is a U.S.-based partnership between Merck (known as MSD outside the United States and Canada) and Sanofi.

§ PRP-OMP, PRP-T, PRP-T, DTaP-IPV/Hib, and DTaP-IPV-Hib-HepB.

¶
https://www.cdc.gov/vaccines/acip/recs/grade/hib-aian-infants-etr.html

**
https://www.cdc.gov/vaccines/acip/recs/grade/hib-aian-infants.html

†† Serious adverse events were defined as hospitalization, death, life-threatening drug experience, or prolongation of hospitalization.

§§
https://www.cdc.gov/vaccines/hcp/imz-schedules/child-adolescent-catch-up.html?CDC_AAref_Val
